# Patterns of Urban Violent Injury: A Spatio-Temporal Analysis

**DOI:** 10.1371/journal.pone.0008669

**Published:** 2010-01-13

**Authors:** Michael Cusimano, Sean Marshall, Claus Rinner, Depeng Jiang, Mary Chipman

**Affiliations:** 1 Injury Prevention Research Office, St. Michael's Hospital, Toronto, Canada; 2 Department of Surgery, University of Toronto, Toronto, Canada; 3 Department of Public Health Sciences, University of Toronto, Toronto, Canada; 4 Department of Geography, Ryerson University, Toronto, Canada; 5 Li Ka Shing Knowledge Institute, St. Michael's Hospital, Toronto, Canada; Swiss Paraplegic Research, Switzerland

## Abstract

**Objectives:**

Injury related to violent acts is a problem in every society. Although some authors have examined the geography of violent crime, few have focused on the spatio-temporal patterns of violent injury and none have used an ambulance dataset to explore the spatial characteristics of injury. The purpose of this study was to describe the combined spatial and temporal characteristics of violent injury in a large urban centre.

**Methodology/Principal Findings:**

Using a geomatics framework and geographic information systems software, we studied 4,587 ambulance dispatches and 10,693 emergency room admissions for violent injury occurrences among adults (aged 18–64) in Toronto, Canada, during 2002 and 2004, using population-based datasets. We created kernel density and choropleth maps for 24-hour periods and four-hour daily time periods and compared location of ambulance dispatches and patient residences with local land use and socioeconomic characteristics. We used multivariate regressions to control for confounding factors. We found the locations of violent injury and the residence locations of those injured were both closely related to each other and clearly clustered in certain parts of the city characterised by high numbers of bars, social housing units, and homeless shelters, as well as lower household incomes. The night and early morning showed a distinctive peak in injuries and a shift in the location of injuries to a “nightlife” district. The locational pattern of patient residences remained unchanged during those times.

**Conclusions/Significance:**

Our results demonstrate that there is a distinctive spatio-temporal pattern in violent injury reflected in the ambulance data. People injured in this urban centre more commonly live in areas of social deprivation. During the day, locations of injury and locations of residences are similar. However, later at night, the injury location of highest density shifts to a “nightlife” district, whereas the residence locations of those most at risk of injury do not change.

## Introduction

Descriptive epidemiology traditionally concentrates on the characteristics of person, time and place. Studies of injury have often concentrated on personal characteristics (for example age, sex, blood alcohol level) and have paid less attention to characteristics of either the time or the place of injury occurrence [1], [2]. While some studies of injuries in traffic collisions have involved time or place, fewer studies of intentional injury have done so [3], [4]. There are few studies on the location of injuries [5].

Violence has been an area of interest in regards to examining the place of injury, since violent injuries are of interest as a legal issue [6], [7]. Nelson et al. examined the geographic incidence of violent crime (not injury related to violence) in the central cores of two British cities using geocoded crime incidence data. Reported violent crimes were most common in areas with high concentrations of drinking establishments, particularly in evening time periods. Central shopping districts, which contain retail stores, restaurants and entertainment venues, also had high clustering of violent crimes. That study identified a pattern of afternoon and early evening violent crime associated with the poor, attributed to “vagrants and drunks [8]”.

Gruenewald et al. conducted a study using hospital discharge data from the State of California and compared assault rates in the population aged 15 and older to alcohol outlets (retail stores, bars and restaurants) and indicators of social deprivation at the zip code level [6]. The authors used the locations of patient residence addresses. Population characteristics and the location of retail alcohol outlets in urban and rural areas were correlated with assault injury discharges. The study found that a concentration of bars did not necessarily result in increased assault injury, as bars were also found in areas with middle to high income levels and low assault rates, and that violence in bars may be determined by their particular clientele [6]. Kim and Pridemore, using epidemiologic techniques, also found strong correlation between violence (in this case, homicide in Russia) and social deprivation, including poverty, family instability and neighbourhood instability in a national-level study [7].

These studies chiefly used home locations of victims in their analyses. Studies using a location of injury could add to our understanding of the space dimension of violence-related injury. However, understanding the dimensions of where injuries occur can be difficult when using data such as emergency room, hospital discharge or trauma registry data, since location of injury is rarely recorded by health care personnel who are the primary source of this data. Although hospital or emergency room registries often record a place of residence, these datasets lack important information about the actual location of injury. This is a clear shortcoming of prior work in this area.

There have been no prior studies that have used only ambulance data to examine the location of injury. Ambulance data provides a measure of the location of injury in addition to a measure of when an injury occurred. If residents of a geographic area are served by a single emergency ambulance system, then such data becomes representative of all ambulance calls of the population under interest. A study of ambulance data in Singapore also found that a very high percentage of ambulance patients as compared to ambulatory patients were those suffering from trauma, particularly those in traffic-related and home-related incidents [9]. A recent study of ambulance records in Sweden has found that ambulance data is useful for regular surveillance of moderate and severe injuries [10]. These studies suggest that ambulance data may be a rich source of location-related data in studying injury. Although trauma registries can provide rich information on the clinical condition of a patient or the patient's response to therapeutic interventions, they often lack any description of the site of injury, since busy emergency department clinicians rarely ask about this information. In contrast, ambulances are routinely sent to precise locations of injured persons and provide precisely the information we need to better understand the spatial aspects of injury. In this study, we used an ambulance dataset that is population-based, precisely geo-coded and time-stamped to provide unique information about the “where” and “when” of injury.

Canada's largest city, Toronto, with a population of 2.4 million and an area of 630 square kilometres, has a single ambulance service serving the emergency needs of its entire population. The data arising from a call to the service is precisely geocoded by longitude and latitude and time-stamped based on the site and time to which ambulances are dispatched. We used the Emergency Medical Services (EMS) dataset of Toronto to study where and when injuries occurred.

To better understand where the victims of injury lived, we used a dataset of all persons attending an emergency department, the National Ambulatory Care Reporting System (NACRS), that has information on the location of a patient's residence to define the location of the victim's home. Each dataset is complementary to the other; however, privacy controls prevented us from linking the two datasets. Both datasets, as is common in Canadian health data, only contain the most basic of demographic, typically just age and sex. Characteristics such as income, ethnicity, immigrant status, and education are not collected in the EMS or NACRS datasets so we incorporated these in an ecological approach using data from the Canadian Census. Maps were created for these indicators of social and ethnic status and were used, in this case, for comparative purposes.

In this study, we used Geographic Information Systems (GIS) to map the specific locations of injury. GIS provides a powerful tool for injury prevention research through its ability to display geographic information and its ability to combine multiple databases to identify specific patterns and their determinants [11]. The purpose of our study was to use the Toronto EMS and the NACRS datasets within a geomatics framework to describe where and when violence-related injury occurred in a large cosmopolitan city.

## Methods

The Toronto EMS is a single provider dataset with approximately 425,000 calls annually. It is fully computerised and uses the Medical Priority Dispatch System, a widely used emergency call sorting algorithm to classify calls. It contains data on the date, time, location (presented as an address or intersection and with latitude and longitude of the location), the reason for the dispatch (simple categorical), the hospital the ambulance was sent to upon receiving the patient, procedures and medication administered on the way, and staffing and vehicle information.

This study utilised all injury-related ambulance dispatches (n = 71,240) to a location in the City of Toronto, Canada, for the years 2002 and 2004. The Toronto EMS dataset uses generalized categories to describe the reason for the ambulance dispatch. Examples of categories include “Stroke,” “Motor Vehicle,” “Fall,” or “Assault.” We included any assault-related injury caused by a gun, knife, sharp or blunt object, or body part (for example a fist). We excluded all calls about patient transfers, cardiac emergencies as well as other non-injury emergencies. We excluded all self inflicted injuries as well as those caused by falls, and those related to traffic, sports, recreation, workplace, poisoning or burns. We also removed duplicate calls or incomplete calls (an ambulance sent but did not transport a patient to a hospital) or those missing age or time data. Duplicate calls or missing data made up approximately three percent of the whole Toronto EMS database. Data for the year 2003 were not available due to a change in the database system, affecting the reliability of the records.

We recorded the time of day at which the dispatch occurred to determine the temporal pattern over a 24 hour period. The Toronto EMS database's only geographic attribute is the location of where the injury occurred. This information is geocoded using each dispatch location's latitude and longitude based on a street address or an intersection of two streets, and is therefore point data that can be mapped. We mapped these geocoded points using ArcGIS ® 8.2. Using the kernel density function to calculate the overall density of the points, we developed density maps of violence-related injury. The kernel density function calculates the density of the points across a moving processing window defined by a bandwidth parameter and assigns density values to cells in a new raster file. One is able to assign values or weights to individual points in a kernel density function. However, in this analysis, we gave all assault cases equal weight, and each represent the direct concentration of assault incidents in the City of Toronto.

The advantage of the kernel density function is that it is a very effective visualization tool for map making and comparing maps with each other. These maps display areas with a large number of points (or areas of high density) especially well without point overlap from a simple point-based map, and also serve to blur the exact locations of injury, thereby addressing patient privacy concerns. The maps show this density as “hot” (high density) and “cold” (low density) spots, using a similar scale as previous authors [12]. We developed these maps for all assault-related injuries over a 24-hour period and then created a set of maps for four hour epochs throughout the day to examine the spatio-temporal changes in patterns of injury over the period of a day. We used a “search radius” (bandwidth) of 0.01 kilometres for an output cell size of 0.0001 kilometres after experimentation with different settings.

Census data was obtained from the 2001 Census of the Population at the census tract (CT) level. The CT is a small level of geography for urban agglomerations and is defined by Statistics Canada for dissemination of Census data and other statistical products. There are 522 populated CTs in the City of Toronto, ranging in size from 0.07 to 28.72 square kilometres, with populations ranging from approximately 1,000 to over 8,000. The Census includes important family, socioeconomic, ethnic and immigration data. The Canadian Census is administered every five years.

Injury counts were also aggregated to the 140 neighbourhoods identified by the City of Toronto's Neighbourhood Profiles. These neighbourhoods were composed of two or more CTs with an average population of approximately 7,000 to 10,000, and they were designed to be cohesive spatial units, taking into account barriers and relative socioeconomic homogeneity [13]. These neighbourhoods are used to organize land uses and socioeconomic variables to explain violent injury hot spots. We mapped and compared local indicators of social deprivation to densities of assaults. These variables, taken from the 2001 Canadian Census, included mean household income and unemployment. In addition, we mapped the locations of social housing complexes and homeless shelters.

The NACRS dataset consists of over 2,000,000 records of people attending an emergency department in the Province of Ontario, Canada, per year, and includes each patient's address determined by their six-digit Canadian postal code. The NACRS includes diagnosis codes (ICD-10), time of the patient's arrival in the emergency department, and the age and sex of each patient. Because of privacy restrictions, the NACRS dataset is not released with full patient address or a full 6-digit postal code. Instead, we linked each record to the CT of the patient's residence location using a postal code-to-census tract conversion file.

Table 1 explains the general similarities and differences between the NACRS and EMS datasets.

**Table 1:** Selected characteristics of EMS and NACRS datasets.

	EMS	NACRS
**Years used**	Jan 2002-Dec 2002, Jan 2004-Dec 2004	Apr 2002 - Mar 2004
**Geographic Identifier**	Place of ambulance dispatch	Address of injured patient
**Geographic Criteria**	All ambulance dispatches within the City of Toronto	All Ontario hospital emergency department visits by Ontario residents
**Lowest level of geography available**	Latitude and longitude (address/intersection)	Census tract
**Diagnoses**	Categorical (Assault, Fall, Traffic, Fire/Burns, Positioning, Sport/Recreational, Self-wound, Other)	ICD-10 (X85-Y09)
**Number of all types of injuries during study**	140,000	260,000
**Violent injuries in ages 18–64 in study**	4,587	10,693

To compare the location of ambulance pick-ups and patient residences, we created kernel density maps based upon the residential CT of patients seen in an emergency room for an assault related injury between the ages of 18 and 64 inclusive for the 2002–2003 and 2003–2004 calendar years, closely approximating the period of time covered by the EMS dataset. The NACRS dataset is coded using ICD-10 codes, so we identified assault injuries by an entry in the range between X85-Y09 inclusive. Kernel density maps for the EMS data were based on the latitude and longitude of the ambulance dispatch location, while the maps for the NACRS data were based on the centroids of CTs with the number of injured used as the population field.

We also created maps representing population density at the CT level to explore patterns seen in EMS and NACRS kernel density maps using point data. To account for population density, EMS and NACRS data was aggregated to the CT level and then an incident rate per 1,000 people in the study population (obtained from the Census) was calculated. We calculated rates of ambulance dispatches based on the numbers of adults living in the CT to which the ambulance was dispatched (EMS dataset). We based rates of hospital admission on the numbers of persons living in the same CT who attended the emergency room and were captured in the NACRS dataset. We then mapped these rates using a standard choropleth method based on CTs.

To further examine the effects of population density on spatial and temporal patterns of assaults, we also fit multivariable models using Poisson regressions. The multivariable analyses were built using the number of assaults at the CT level for all time periods as dependent variables. These dependent variables were discrete and had limited count values. Exploratory data analyses indicated that the distribution of these dependent variables was positively skewed and followed approximately the Poisson distribution with potential over dispersion. Therefore, we fitted the Poisson regression model for each of these dependent variables. The models were estimated by the maximum likelihood method, and the effects of the independent variables were tested by Wald tests. The independent variables included in the regressions are the number of bars, the working age population (in 1 000 s), the number of social housing complexes, the unemployment rate and average household income. The residuals from these regression analyses were output to plot in GIS software to examine whether the spatial (or temporal) effect after controlling for these factors still exists.

## Results

In the Toronto EMS database, all types of injury-related calls accounted for 71,000 (25.8 percent) of a total of approximately 260,000 ambulance dispatches per year. There were 4,587 (2,071 in 2002 and 2,516 in 2004) ambulance dispatches for assaults for interpersonal violence related injuries for those aged 18 to 64 (population aged 18–64 was 1,624,220) in the years 2002 and 2004, or an annual rate of 141.21 per 100,000 persons. In the NACRS database, there were 10,693 assault injuries recorded, or 16.3 percent of all 65,508 emergency room visits for all injuries over 2 years. This works out to an annual rate of 329.17 assault hospitalizations per 100,000 persons. There is an obvious difference between the number of EMS and NACRS records. As there is an overlap of time periods, we checked, and confirmed that the numbers are not significantly different even when only examining the 12 months (April 2002 to December 2002 and January 2004 and March 2004) which the two datasets overlap.

### Temporal Pattern of Violent Injury

**Figure 1:**
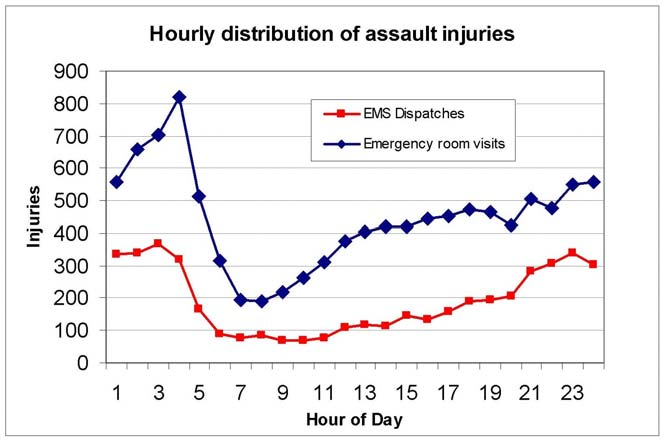
Hourly distribution of assault injuries in Toronto.

The 24-hour temporal patterns of all assault-related injuries for the city in the EMS and NACRS datasets are shown in Figure 1. This clearly shows a temporal pattern with a peak of violence-related injuries occurring at night with a nadir in the morning followed by a slow rise during the afternoon. Two specific four-hour time periods were particularly notable. In the EMS dataset, the time periods between 20:00 and 23:59 accounted for 26.2 percent of all assaults and the time period between 0:00 and 3:59 accounted for 26.8 percent of all cases. We found similar results with the NACRS dataset, where the period between 20:00 and 23:59 accounted for 19.5 percent of all cases, and the period between 0:00 and 3:59 accounted for 25.6 percent of all assaults.

### Spatial Distribution of Violent Injury

**Figure 2:**
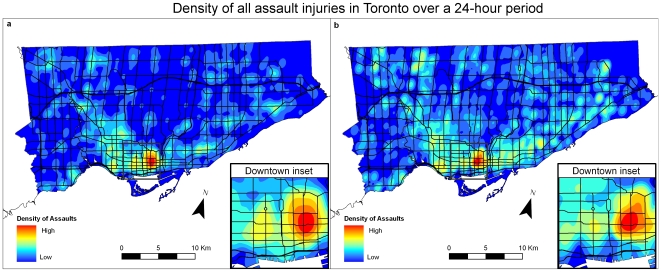
Assault injury densities in Toronto using data aggregated for 24 hours. Figure 2a: Density of all assault injuries for EMS dataset over a 24-hour period. Figure 2b: Density of all assault injuries for NACRS dataset over a 24-hour period.

Figure 2 shows the spatial distribution of assault injuries aggregated over a 24-hour period for location of injury (EMS dataset) and home locations of the injured people attending emergency departments (NACRS dataset). Figure 2a, a density map for assault related calls in the EMS database, shows that the highest concentration (shown as red-coloured areas) is in the eastern downtown area. Areas of moderate density (shown by a yellow-to-green shade) are located in a west-end neighbourhood, as well as a few midtown locations to the northwest and east of the core. Otherwise, there are a few areas of moderate injury density with a “U” shaped pattern as these areas spread from the downtown core to the northwest and northeast.

Figure 2b is a density map of assaults aggregated over 24 hours using the NACRS dataset for emergency department visits. This pattern is similar to that seen with the ambulance data – the east downtown hot spot is located in approximately the same place as with the EMS dataset, and the citywide “U” shaped pattern is even more obvious than with the EMS data.

### Spatio-Temporal Patterns of Assault-Related Injury

**Figure 3:**
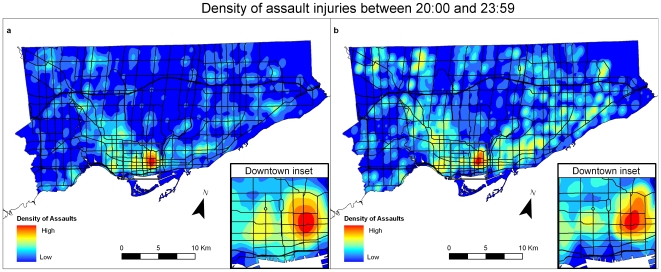
Assault injury densities in Toronto using data aggregated for 4 hours (20:00 and 23:59 hrs). Figure 3a: Density of assault injuries between 20:00 and 23:59 for EMS dataset. Figure 3b: Density of assault injuries between 20:00 and 23:59 for NACRS dataset.

A series of distinctive spatial patterns occurred when we explored the patterns over four-hour epochs. During daytime hours, the patterns seen in the EMS and NACRS datasets were similar. For example, Figure 3 shows that in the early evening, the area of the greatest concentration of assault injuries is similar to that seen in the 24-hour aggregated map. This area is characterised by a high proportion of social housing communities, homeless shelters, high unemployment rates and a very low mean household income (Figures 5a through 5c).

**Figure 4:**
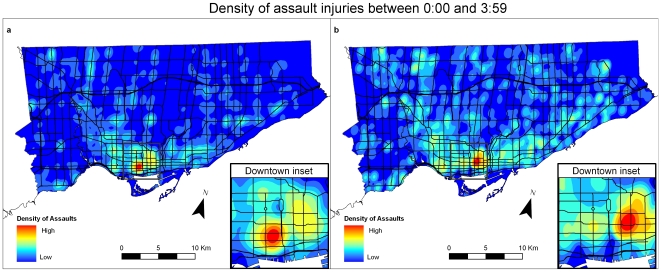
Assault injury densities in Toronto using data aggregated for 4 hours (00:00 and 03:59 hrs). Figure 4a: Density of assault injuries between 00:00 and 03:59 for EMS dataset. Figure 4b: Density of assault injuries between 00:00 and 03:59 for NACRS dataset.

**Figure 5:**
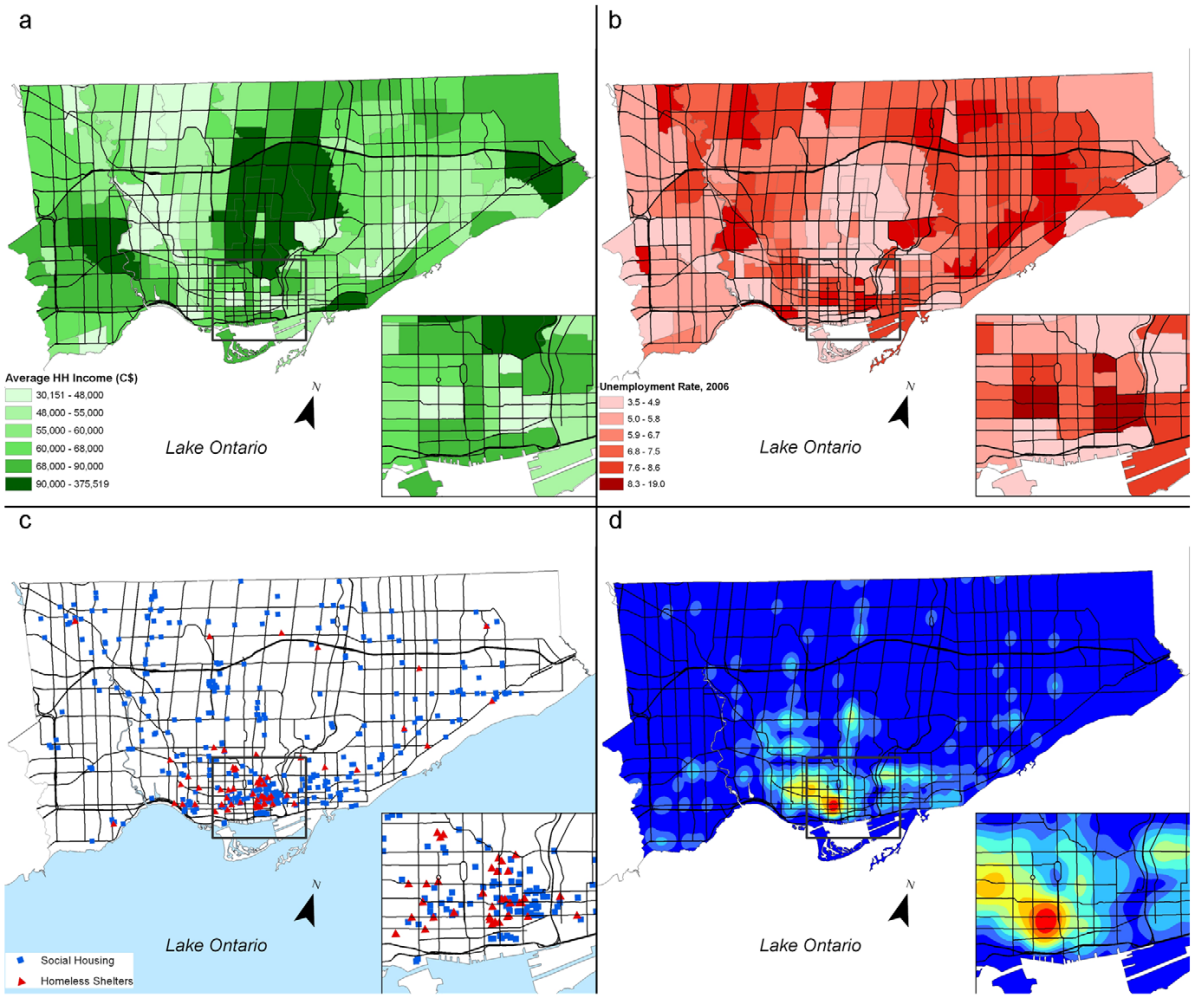
Land use and economic characteristics of the City of Toronto (2001 Canadian Census). Figure 5a: Mean household income. Figure 5b: Unemployment rate. Figure 5c: Social housing and homeless shelters. Figure 5d: Density of alcohol drinking establishments.

In the early morning time period, from 0:00 to 3:59, the most prominent “hot spot” for ambulance dispatches for assault injuries shifts to a southwest downtown area, known as the “Entertainment District” (Figure 4a). This area is characterized by relatively few residents with a high mean household income and a high density of bars and clubs (see Figure 5d). The time period with the highest counts of injuries corresponds to the time just after official closing time for establishments licensed to serve alcohol (02:00). In contrast to the pattern seen with the location of injury (EMS dataset), no similar change occurs in the location of residences of those admitted to emergency departments for assault (Figure 4b).

**Figure 6:**
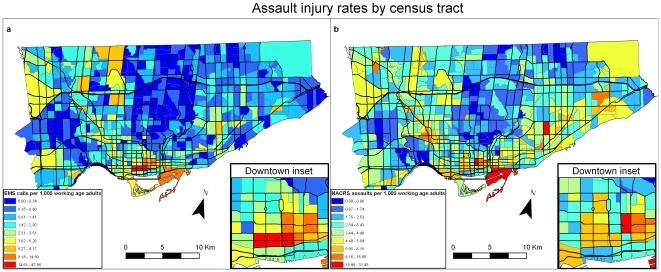
Choropleth maps of rates of assault in injury by census tract (2001 Canadian Census). Figure 6a: Rates of injury for assaults for EMS database by census tract. Figure 6b: Rates of injury for assaults for NACRS database by census tract.

To account for population density, EMS and NACRS data was aggregated to the CT level and then an incident rate per 1,000 persons in the study population was calculated. Figures 6a and 6b show the rates of ambulance dispatches for assaults and emergency room visits for assault injuries over all time periods. These maps illustrate that the patterns of assault injury controlled for population density are similar to the pattern of density of assaults (Figure 2a, 2b).

The results of the multivariate analyses are shown in Tables 2 and 3. The results show significant effects for population density, the number of bars, social housing complexes, and mean household income. The unemployment rate was a significant predictor for emergency room visits but not ambulance dispatches. Maps of the residuals from the multivariate models confirmed these findings.

**Table 2:** Poisson Regression Estimates of Number of EMS Assaults.

	Total EMS assaults		EMS assaults between 20–24PM		EMS assaults between 0–4AM	
	Estimate(SE)	p-value	Estimate(SE)	p-value	Estimate(SE)	p-value
Intercept	1.45(0.20)	<.001	0.26(0.24)	0.281	0.36(0.25)	0.149
Adults population (in 1000)	0.19(0.03)	<.001	0.21(0.03)	<.001	0.22(0.03)	<.001
Number of bars	0.09(0.01)	<.001	0.07(0.01)	<.001	0.11(0.01)	<.001
Number of Social Housing Complexes	0.14(0.02)	<.001	0.14(0.03)	<.001	0.13(0.03)	<.001
Unemployment rate	0.02(0.02)	0.143	0.02(0.02)	0.284	0.01(0.02)	0.823
Average household income (in 10000$)	−0.08(0.03)	0.005	−0.12(0.04)	0.002	−0.13(0.04)	0.002

Note: 1. SE: standard error of estimate.

**Table 3:** Poisson Regression Estimates of Number of NACRS Assaults.

	Total NACRS assaults		NACRS assaults between 20–24PM		NACRS assaults between 0–4AM	
	Estimate(SE)	p-value	Estimate(SE)	p-value	Estimate(SE)	p-value
Intercept	2.52(0.11)	<.001	1.16(0.16)	<.001	1.03(0.15)	<.001
Adults population (in 1000)	0.22(0.01)	<.001	0.22(0.2)	<.001	0.22(0.02)	<.001
Number of bars	0.05(0.01)	<.001	0.03(0.01)	0.004	0.05(0.01)	<.001
Number of Social Housing Complexes	0.12(0.01)	<.001	0.09(0.02)	<.001	0.12(0.02)	<.001
Unemployment rate	0.02(0.01)	0.020	0.02(0.01)	0.050	0.02(0.01)	0.030
Average household income (in 10000$)	−0.15(0.02)	<.001	−0.22(0.03)	0.002	−0.12(0.02)	<.001

Note: 1. SE: standard error of estimate.

## Discussion

Most injury studies in the literature use single hospital-related or health system data. These datasets usually include patient residence rather than injury location since this data is more easily collected in hospital or administrative databases. In contrast, the Toronto Emergency Medical Services dataset is based on the location where each injured patient was picked up by the ambulance team. As a result, the EMS database provides a good approximation of where serious injuries occur. In addition, since there is only a single emergency ambulance transport provider in Toronto, this study provides, to our knowledge, the only population-based study of the spatial-temporal patterns of assault-injury locations occurring in a large urban setting.

Aggregated over a 24-hour time period, our results are similar to those of Sampson, Raudenbush and Earls, who used Census and survey data to measure neighbourhood characteristics and violent crime rates for Chicago's 343 neighbourhoods [14]. Survey questions in that study included perceived levels of crime and community response to crime and antisocial behaviour, while Census data were used to measure social deprivation. The authors found that neighbourhood stability, social deprivation, and local immigrant concentration explained 70 percent of variation in the perception of crime and community response and violent crime rates [14].

One of the most interesting temporal variations that we discovered in this study is that spatial patterns of urban assault-related injury are not static, but rather dynamic. There are two distinct high-risk locations which may relate to different populations, or the same population that moves with time. The first high risk area, the downtown east side, is closely related to several indicators of social deprivation as seen above in Figure 5. There is also a very close association to areas of high densities of night clubs and bars that are prominent at night. These different locations may relate to different populations or the same population that moves with time. The very close association to areas of high densities of night clubs and bars in the night and early morning likely suggest that the at-risk population is engaging in risk-related activities such as alcohol and drug use. The two highest hourly counts for assault injury dispatches in the EMS database occur between 22:00 and 22:59 and again between 2:00 and 2:59. The periods between 20:00 and 23:59 and between 0:00 and 3:59 account for more than half of all assault-related injuries. Our results show a distinctive geographic shift that occurs in injury location to an area characterized by a high number of bars and clubs and few residents, and this shift coincides with the closing time for alcohol-licensed establishments. Opening times for bars and clubs vary – many pubs, for instance, open for the lunch period, but nightclubs, which are especially concentrated in the “Entertainment District” open much later, starting at 20:00. These findings point to behavioral and social factors in the immediate environment that may be associated with these shifts in injury occurrence. The shift to night-life districts has implications for preventive interventions such as policy, law enforcement and strategies aimed at alcohol and drug use.

These findings are particularly interesting as there is no discernable temporal shift in the pattern of patient residences as shown in maps from the NACRS database. What we find is while the location of assault injury changes, especially at night and the early morning, the hot spots of location of residences does not.

Other authors have demonstrated the concentration of violence and disorder to relatively small city centre entertainment districts, most frequently in and around bars and clubs on weekend nights [15]. Nelson et al., who worked in Cardiff and Worcester in the UK, also showed major clusters of violent crime at night in the pub/leisure zones of the city centre and secondary clusters during the day in major retail streets [7]. Sherman, Gartin, and Buerger showed that nearly 50 percent of calls to police for violent crime related to 3 percent of locations in Minneapolis and “hot spots” emerged in every category of crime suggesting that certain places were “criminogenic [16]”.

Hope found that in the British city of Newcastle-Upon-Tyne, 42 percent of crimes occurred on four streets that had 15 percent of public houses [17]. Warburton and Shepherd hypothesised that the concentration of alcohol premises in close proximity to each other and the presence of high-risk premises leads to violence [18]. Our work shows that these areas shift over the course of a day and these findings have implications for planning preventive interventions.

Our study demonstrated that the shifts were from areas of relative social deprivation during daylight and early evening hours to areas of higher income, lower residential density and higher densities of drinking establishments. Privacy and other restrictions of our data prevent us from determining whether individuals from the same areas are being injured or whether these are persons migrating to these areas that are being injured. In a case-control study conducted in Bristol, UK, Shepherd, Robinson, and Levers showed that the victims of city-centre violence treated in an emergency department between 23:00 and 3:00 on weekends were associated with excessive binge-alcohol consumption, but not with alcohol dependence, unemployment at the time of violence, or socioeconomic group [19]. Our study demonstrated an association between closing time of alcohol establishments and the incidence of assault. Hope and Nelson et al. found similar patterns of crime occurring around the closing time of these establishments [17], [7]. Graham, Bernards, Osgood, and Wells [20] studied large drinking establishments and found that the immediate bar-room environment has an important impact on the frequency of aggression but less so on the severity of aggression. The strongest predictors of both the frequency and severity of patron aggression in large drinking establishments were social factors such as rowdiness/permissiveness and sexual activity, contact and competition, and closing time variables such as over-serving at closing time and the number of people hanging around after closing.

Understanding the “where”, “when”, “who”, and “how” of injury is essential in developing strategies for all phases of prevention and control. The ability to map health data, identify and formulate hypotheses about spatial patterns, and build models makes the geomatics framework used in this study a powerful tool for public health, health policy research, and epidemiology. This study is one example of how geomatics can help to clearly identify and see spatial and temporal patterns in health-related data.

### Limitations

A limitation of our study may be that of underreporting of assault-related injury due to a reluctance to report to authorities. Nelson et al. note that underreporting of assaults was common in a British study of violent crime – while just over 30 percent of common assaults were reported, 60 percent of woundings are reported [7]. Our EMS data would not include cases in which patients are brought directly to an emergency department without ambulance aid. In fact, our NACRS dataset is almost twice the size of the EMS datasets, suggesting that each may include patient characteristics that differ from each other. For example, the NACRS data may include patients who walk in to the emergency or may have been brought to the emergency room from a scene of injury. Our NACRS data would not include those persons who attend other facilities such as family doctor's offices and local health clinics.

Most emergency department data, including NACRS, do not contain detailed information surrounding the location of injury, apart from perhaps a basic categorical indicator such as “home” or “on street.” Although our EMS data may not represent the actual location of injury but rather the location that the patient was picked up by the ambulance crew, the two locations will likely be close to each other, especially for those more seriously injured and thus needing an ambulance for transport to the hospital. Ideally, we would have linked our two datasets; however, privacy laws and research ethics board considerations prevented us from doing this. Despite this limit, we believe the data still provide a valuable geocoded population-based measure of injury location that has rarely been used before.

There are some cautions necessary when comparing the respective locations of assault injury using one dataset and patients using a second dataset. While all persons brought in by ambulance and hospitalized would be found in both datasets, there is not a perfect match. Some ambulance patients will live outside of the City of Toronto, including suburban residents and visitors. Some persons in the NACRS dataset may have been injured outside of Toronto. Without common identifiers in both datasets, and due to privacy issues noted above, the two datasets unfortunately cannot be linked at the present time. Although a Swedish group [10] has collected residence addresses in ambulance data, the urgency of the ambulance scenario makes it unlikely that even this dataset would contain the richness of data possible by accurately linked emergency room, hospital and ambulance data.

Our study, like any geographic study, is prone to the “ecological fallacy” that occurs when assumptions are made about individuals based on aggregated data. For instance, when associating particular socio-economic characteristics with injury risk factors (for instance, low income), it cannot be assumed that individuals living in (or, in this case, injured in) high-risk areas are directly represented by the general characteristics of those areas.

We also calculated “rates” of ambulance dispatches for assault injury based on the number of people living in the CT from where the patient was picked up by the ambulance service. A better denominator may have been the number of people present in an area at the particular time that the dispatch occurred. For example, a small number of assaults occurring during a sporting event in a CT that contains a large sports stadium next to a few homes may have high rates of injury. If one considers the rates by the numbers of people visiting that location rather than the number of residents in that location, different conclusions may occur. Our work and noted rates with entertainment districts might be susceptible to this phenomenon.

Another limitation relates to those persons without a specific location or time of dispatch. In our database, approximately 1.6 percent of all ambulance calls had missing time or location data but only 0.1 percent of assault entries lacked a specific time or location code. This indicates that injuries caused by assaults may be well represented within ambulance datasets due to their particular nature.

Although our analysis can display associations of community-based data with injury data, it is important to understand that, in and of itself, our study cannot infer causality. However, the use of ambulance data to examine the time and place of injury can be very useful as an epidemiological tool. Studies like the one presented here can provide a rational basis for causality; it can assess changes over time and risk exposure, and also be used as an analytical tool before and after interventions. It can also be used in a predictive capacity to help understand how the present state might change with interventions directed at changes to community-based risk factors.
